# Psychiatry (3rd ed.) by Neel Burton, MD

**DOI:** 10.5964/ejop.v12i2.1182

**Published:** 2016-05-31

**Authors:** Beatrice Popescu

**Affiliations:** a EJOP Founding Editor; University of Bucharest, Bucharest, Romania

[Other p1]*Psychiatry*, the new textbook by Neel Burton, philosopher, psychiatrist and writer as he loves to present himself to its newest readership, is not the typical manual a student or a resident in psychiatry is used to. The author, seduced forever by the art of psychiatry as much as by the art of philosophy, is therefore challenged to make the discipline more accessible, interesting and vibrant to the students eager to delve into the hottest specialism on the market, considered not long time ago the ‘Cinderella’ of healthcare.

Trying to break away from the arid and scholastic DSM-5 style, especially via the introductory chapter, the history of psychiatry, Neel Burton invites the reader to indulge himself into a cultural travel through ages, from Ancient Greece through Enlightenment to the modern era. Chapter 2 is dedicated to the psychiatric assessment or the anamnestic interview, the most important stage before reaching a diagnosis. What distinguishes this manual from a classical standard psychiatric manual are the text boxes that teach clinical skills, using open, closed and leading questions and also the genogram, usually one of the diagnostic tools of psychotherapy when assessing genetic determinism or early schemas. Towards the end of the chapter, a thorough sample psychiatric assessment is presented, while the etiology is explained in a tripartite description. Chapter 3 which is an overview of the UK mental health community care system contains a so much needed ethics section, backed by an interesting case study and also by a so much useful part with the UK Mental Health Act explained. A very brief overview of the most important types of therapies follows soon.

Part two of the manual is entirely dedicated to the plethora of pathologies, the heart and soul of psychiatry, from schizophrenia to sexual disorders, but in a manner slightly different from the DSM or ICD arid, unengaged and pathologizing and labeling style.

Perhaps one of the most fascinating chapters in the history of psychiatry and also in the history of philosophy is suicide. Albert Camus is stating in *The Myth of Sisyphus* that "there is but one truly serious philosophical problem and that is suicide". The suicide complex phenomenon is comprised by the author in Chapter 6 of his manual, *Suicide and deliberate self-harm*. A large introduction in the suicide ethics quoting David Hume, Satre and Pliny the Elder prepares the reader for the philosophical assumptions behind choosing death over life. A more didactic approach of assessing suicide risk factors and management follows and the chapter closes with listing useful clinical skills when counseling people with self-harm thoughts. Perhaps the extensive clinical skills section is highly justified, considering the self-harm and suicidal behaviors the scariest ones for a mental health professional when dealing with a patient or client. The mental health professional is therefore fully equipped, at least theoretically, with the clinical skills required when faced with this sort of dramatic and challenging theme.

In Chapter 7, a special attention is given to the anxiety, stress related and somatoform disorders especially from a psychosocial perspective, and also a philosophical cure for anxiety following Maslow’s pyramid of needs is provided. According to the existential outlook of anxiety, also shared by Paul Tillich, Sartre, Camus and Yalom, the author of this book also supports the view that pathological anxiety, although grounded in threats of life, in fact arises from repressed existential anxiety. In a way, Neel Burton seeks a cure for anxiety in the same manner as Reid Wilson^i^ does: if the ultimate source of anxiety is uncertainty, then the cure itself proposes to seek out uncertainty while accepting death as the only certainty. However, fear of death should be able to make us seek "the most out of our lives and out of ourselves", concludes the author.

Personality Disorders, described extensively in chapter 8 of this fascinating textbook are those character disorders firstly coined by JC Pritchard as "moral insanity" behaviors to the Kraepelin’s "psychopatic personality" disorders. The author stresses the fact that not only characterizing the personality disorders is a difficult task, but also diagnosing them. We would add that treating them seems even tougher, especially since there are patterns relatively stable across time and consistent across situations. The particularity of Neel Burton’s approach is seeing personality disorders as possible routes to high professional achievements. The author thinks that while personality disorders may lead to "severe impairment", they may also lead to extraordinary achievements. Quoting the Board and Fritzon study, Neel Burton describes the executives with a personality disorder as "successful psychopaths", while criminal offenders are seen as "unsuccessful psychopaths". In the same chapter a complete review of ego defense mechanism follows, revealed in a psychoanalytical tradition and also from the Hindu Bhagavad Gita viewpoint, the latest adding the flavor of originality to the ‘orthodox’ view.

Chapter 11 is dedicated to a profound understanding of the substance misuse (alcohol and drugs) reasons behind. The blue butterfly box reveals the psychological causes of delving into such a behavior by quoting a character from *Trainspotting*, Renton. This draws me very close to an idea possible to implement when trying to present the perils of starting smoking marijuana to the high-school teenagers by offering them to watch a shortcut version of Aronofsky’s *Requiem for a Dream*. The author believes that rather than employing a cold outlook when presenting a sufferer the dangers of using alcohol and recreational drugs, a mental health professional should actually use motivational interviewing techniques which he exposes in the clinical skills section (p. 154).

Dr. Neel Burton’s complex anthropological, psychiatric, philosophical and psychological approach when designing *Psychiatry* textbook makes the student’s job very enjoyable and captivating while preparing for exams. Not only students or residents in psychiatry could benefit from his fresh and lively approach, but also other professionals: clinical psychologists, psychotherapists, general practitioners in their effort to screen mental disorders and also sociologists and anthropologists. The profound humanistic view and storytelling approach drawing slightly further from the canonic style of DSM makes this interesting manual an enjoyable reading and also a powerful professional tool.

